# Supplementary material for: Factors associated with antenatal depression in the Kingdom of Jordan during the COVID-19 pandemic
Source: PLOS Glob Public Health. 2022 Feb 18;2(2):e0000194. doi: 10.1371/journal.pgph.0000194 (PMC10021866; doi:10.1371/journal.pgph.0000194)
Supplement: S1 File — A survey of the knowledge, attitudes and practices of pregnant Jordanian women towards the coronavirus disease (COVID-19) during the outbreak period in Jordan. (PDF) [file pgph.0000194.s001.pdf]

**A survey of the knowledge, attitudes and practices of pregnant Jordanian women towards the coronavirus disease (COVID-19) during the outbreak period in Jordan.**

Dear *Pregnant Mother*,

In light of the spread of the global epidemic of Corona and the exceptional circumstances we are currently living in, there are many pregnant Jordanian women who suffer from difficulty obtaining information and appropriate medical care during pregnancy. This study will help in assessing these difficulties and study appropriate solutions. Therefore, please fill out this short questionnaire, which takes about 10 minutes to complete.

There are no right or wrong answers. The information provided will be used for scientific research purposes only and to derive effective recommendations for improving the health care provided to pregnant women in such exceptional circumstances.

Dear mother, the confidentiality and privacy of the information is preserved, as no information that indicates your personality, such as your name and others, will be taken. Filling out this questionnaire indicates tacit approval to participate in this study and this will not affect you negatively or positively.

With great thanks and appreciation

Dr. Sanaa Abujilban
Faculty of Nursing at the Hashemite University
