# Supplementary material for: Factors associated with antenatal depression in the Kingdom of Jordan during the COVID-19 pandemic
Source: PLOS Glob Public Health. 2022 Feb 18;2(2):e0000194. doi: 10.1371/journal.pgph.0000194 (PMC10021866; doi:10.1371/journal.pgph.0000194)
Supplement: S2 File — (DOCX) [file pgph.0000194.s002.docx]

**Knowledge, attitudes, and practices of Jordanian pregnant women towards coronavirus disease (COVID-19) during the period of its outbreak: Cross-sectional survey**

**دراسة أستقصائية عن معرفة ومواقف وممارسات النساء الاردنيات الحوامل أتجاه مرض فيروس كورونا (COVID-19) خلال فترة تفشي المرض.**

عزيزتي الام الحامل
في ظل إنتشار وباء كورونا العالمي والظروف الإستثنائية التي نعيشها حالياً، هنالك العديد من النساء الاردنيات الحوامل اللواتي يعانين من صعوبة الحصول على المعلومات والرعاية الطبية المناسبة خلال فترة الحمل. وهذه الدراسة ستساعد على تقييم هذه الصعوبات ودراسة الحلول المناسبة. لذا أرجو تعبئة هذه الأستبانة القصيرة والتي تحتاج الى ما يقارب خمس دقائق لإكمالها.
لا توجد أجابه صحيحه أو خاطئه. والمعلومات المقدمة ستستخدم لأغراض البحث العلمي فقط وأستنتاج التوصيات الفعالة لتحسين الرعاية الصحية المقدمة للنساء الحوامل في مثل هذه الظروف الأستثنائية.

عزيزي الام، سرية وخصوصية المعلومات محفوظة حيث لن يتم أخذ أي معلومات تدل على شخصيتك مثل الأسم وغيرها. وتعبئة هذه الاستبانه تدل على الموافقه الضمنية للأشتراك في هذه الدراسه ولن يؤثر عليك هذا بالسلب او الإيجاب.

مع بالغ الشكر والتقدير

**د. لينا مريّان**

**د. سلوى العبيسات**

**د. سناء ابوجلبان**

**كلية التمريض في الجامعة الهاشمية**

**05-3903333 / فرعي (5516)**

**هل انتِ حامل الأن؟**

- نعم
- لا

اذا (**لا)** الرجاء عدم استكمال الاستبيان

إذا **(نعم**) كم عمرالحمل بالاشهر تقديراً؟

1. **المعلومات الديموغرافية والاكلينيكية**
2. **الجنسية:**

- اردنية
- غيرها

1. **العمر:**
2. **الحالة الاجتماعية الحالية**

- متزوجة
- منفصلة فقط – لكن دون طلاق شرعي
- مطلقة
- ارملة

**أين تسكني حاليا ؟ المنطقة _______________ مدينة / قرية ________________**

**طريقة السكن الذي تعيشين فية حاليا؟**

سكن مستقل مع زوجي وأطفالي

سكن مستقل مع أطفالي فقط

سكن مستقل لوحدي

سكن مشترك مع أحد الأقارب مثل الأباء أوالاخوة أو أهل الزوج

غير ذلك

1. **المستوى الدراسي**

- ابتدائي
- ثانوي
- دبلوم متوسط
- بكالوريوس
- دراسات عليا ( دبلوم عالي ،ماجستير ، دكتوراه)

1. **كم سنة قضيت في المدرسة........................سنة**
2. **التخصص الدراسي**

تخصصات علمية و صحية

تخصصات أدبية و أنسانية

لا يوجد – أقل من توجيهي

1. **قطاع العمل**

- حكومي
- خاص
- تطوعي ومجتمعي
- لا أعمل – ربة منزل

1. **بشكل عام, كم معدل ساعات النوم اليومية عندك -----------؟**
2. **هل تدخنين السجائر(اللفائف) أو ألنارجيلة ؟**

- نعم
- لا
- إذا كانت إجابتك نعم كم سيجارة يوميا....................

1. **عمر الزوج: ___________ سنة**
2. **الوضع الوظيفي للزوج؟**

- يعمل بدوام كامل
- يعمل بشكل جزئي
- لا يعمل

1. المستوى التعليمي للزوج:

- ابتدائي
- ثانوي
- دبلوم متوسط
- بكالوريوس
- دراسات عليا ( دبلوم عالي ،ماجستير ، دكتوراه)

**الدخل الشهري بالدينار الاردني :**

**هل لديكِ تأمين صحي؟**

نعم

لا

1. **كم مره حملت ؟ ___________ (احتسبي هذا الحمل مع اجمالي عدد مرات الحمل)**
2. **كم عدد اطفالك الاحياء الان ؟**
3. **هل اجهضت اي من احمالك السابقة**

- لا
- نعم
- أذا أجبت بنعم، كم مرة أجهضت؟

1. **هل كنت تعانين أي مشكلة صحية قبل الحمل:**

- لا
- نعم

1. **أذا أجبت بنعم، اي مشكلة من التاية عانيت:**

- **الصرع**
- **لسكري**
- **أرتفاع ضغط الدم**
- **أمراض قلب**
- **غير ذلك....................**

1. **هل تعانين من اي من الامراض الموسمية مثل الأنفلونزا الموسمية، حساسية الربيع، الحساسية الموسمية الجلدية...الخ)؟**

- نعم
- لا

1. **من بداية حملك هل تم تشخيصُك بأحدى الحالات التاليةِ (أو أكثر من واحده, اختاري الذي يَنطبقُ):**

- لا مشاكل طبيةَ أبدا
- مرض السكّري / مرض سكّري الحمل
- إرتفاع ضغط الدم/ تسمم الحمل / قَبْلَ التشنّج الحملي / تشنّج حملي
- امراض في القلب
- حمل بتوائم
- اجهاضات سابقة
- مشاكل بالسائلِ أو الأغشية المحيطه بالجنين / أَو بالمشيمة
- زيادة في الوزن (سمنة مفرطه)

**هل تراجعي الطبيب بشكل دوري وملتزم بجميع المواعيد خلال فترة الحمل أوما بعد الحمل؟**

نعم

لا

1. **كم كان عدد الزيارات التي قمت بها للأطمئنان على صحة الحمل؟...........................**

**هل تجنبتِ أو قمتِ بتأجيل زيارة الطبيب المقررة لكِ أو لطفلكِ خلال فترة الحجر الصحي وتفشي وباء كورونا؟**

نعم

لا

**أذا نعم، كم مرة زرتي الطبيب خلال فترة الحجر الصحي وتفشي وباء كورونا؟**

1. **مكان زيارة الطبيب؟**

- مستشفى حكومي / عسكري
- مستشفى جامعي
- مستشفى خاص
- عيادة خاصة
- مركز صحي
- غير ذلك

1. **هل قدم لكِ أحد مقدمي الرعاية الصحية معلومات صحية بخصوص فيروس كورونا وتأثيرة على صحتك وصحة طفلك وسبل الوقاية منه خلال فترة الحمل أو ما بعد الولادة؟**

- نعم
- لا

1. **اذا نعم، هل كانت المعلومات التي قدمت لكِ مفيدة بشكل كافٍ؟**

- نعم
- لا
- لا أعلم

1. **من الذي قدم لكِ المعلومات؟**

- طبيب
- قابلة
- ممرضة
- غيره

1. **كيف تم التواصل معك وتقديم المعلومات؟**

- خلال زيارة الطبيب / القابلة / الممرضة
- زارك في المنزل
- عبر اتصال هاتفي
- عبر وسائل التواصل الاجتماعي
- عن طريق البريد الالكتروني
- غيرها

**Assessment of Knowledge**

**حسب معلوماتك الشخصية، أجب على الأسئلة التالية المتعلقه بمرض كورونا :**

1. **المسبب الرئيسي لمرض كورونا هو فيروس؟**

- صحيح
- خطأ
- لا اعلم

1. **المسبب الرئيسي لمرض كورونا هو بكتيريا؟**

- صحيح
- خطأ
- لا اعلم

1. **نقص او ضعف مناعة الجسم تزيد من إحتمالية الأصابة بمرض كورونا؟**

- صحيح
- خطأ
- لا اعلم

1. **مرض كورونا هو مرض وراثي؟**

- صحيح
- خطأ
- لا اعلم

1. **مرض كورونا هو مرض معدي ينتقل من شخص الى اخر؟**

- صحيح
- خطأ
- لا اعلم

1. **ينتقل مرض كورونا عن طريق؟ ( يمكن اختيار اكثر من اجابة تعتقد انها صحيحة)**

- الهواء
- نقل الدم
- رذاذ العطاس
- مخالطة شخص مصاب عن طريق المس أو التقبيل
- استخدام ادوات شخص مصاب
- ينتقل عن طريق الاتصال الجنسي

1. **ما هي علامات الاصابة بمرض كورونا؟ ( يمكن اختيار اكثر من اجابة تعتقد انها صحيحة)**

- ارتفاع الحرارة
- سعال جاف
- تعب عام
- صعوبة في التنفس
- صداع
- أعراض أخرى (احتقان الانف، سيلان الانف، العطاس، أسهالات، خلل في الذوق و الشم)

1. **الاعراض التالية يندر ان تحصل مع مرض كورونا (احتقان الانف، سيلان الانف، العطاس، أسهال)**

- صحيح
- خطأ
- لا اعلم

1. **الى الان, لا يوجد علاج فعال لعلاج الاصابة بمرض كورونا، ولكن العلاج الموجود هو لعلاج أعراض المرض والتخفيف من حدتها.**

- صحيح
- خطأ
- لا اعلم

1. **الكبار بالسن والذين يعانون من نقص في المناعة او لديهم أمراض مزمنة عرضة أكثر للأصابة بمرض كورونا وحصول مضاعفات شديده قد تكون قاتلة.**

- صحيح
- خطأ
- لا اعلم

1. **تجنب الأتصال بالحيونات البرية او المنزلية يقلل احتمال الأصابة بمرض كورونا؟**

- صحيح
- خطأ
- لا اعلم

1. **ينتقل مرض كورونا من شخص مصاب الى أخر سليم اذا كانت أعراض المرض (مثل الحمى) ظاهرة على الشخص المصاب فقط ؟**

- صحيح
- خطأ
- لا اعلم

**ارتداء الكمامات الطبية عن الخروج من المنزل تقلل من خطر الاصابة بمرض كورونا**

- صحيح
- خطأ
- لا اعلم

1. **أرتداء الكفوف البلاستيكية او المطاطية عن الخروج من المنزل تقلل من خطر الأصابة بمر ض كورونا؟**

- صحيح
- خطأ
- لا أعلم

1. **ليس من الضروري للأطفال والشباب اتخاذ إجراءات لمنع الإصابة بمرض كورونا**

- صحيح
- خطأ
- لا اعلم

1. **لتقليل خطر الإصابة بـمرض كورونا، يجب على الأفراد تجنب الذهاب إلى الأماكن المزدحمة وترك مسافة أمان اقل شيء مترين عن الأشخاص الاخرين؟**

- صحيح
- خطأ
- لا اعلم

1. **لتقليل خطر الإصابة بـمرض كورونا، يعد غسل اليدين بالماء والصابون بشكل متكرر بعد ملامسة الاشياء أو الاشخاص او العودة من الخارج الى المنزل ضروري؟**

- صحيح
- خطأ
- لا أعلم

1. **يعد عزل وعلاج الأشخاص المصابين بفيروس كورونا من الطرق الفعالة للحد من انتشار الفيروس.**

- صحيح
- خطأ
- لا اعلم

1. **يجب عزل الأشخاص الذين لديهم اتصال بشخص مصاب بفيروس COVID-19 على الفور في مكان مناسب لمدة 14 يومًا.**

- صح
- خطأ
- لا اعلم

1. **تبدأ علامات وأعراض مرض كورونا بالظهور على الشخص المصاب ؟**

- بشكل مباشر بعد الأصابة
- كحد أعلى خلال أسبوع
- كحد أعلى خلال أسبوعين فقط
- كحد أعلى خلال أسبوعين أو أكثر في بعض الحالات

1. **هل تؤثر الاصابة بمرض كورونا على إمكانية الأرضاع الطبيعي؟**

- نعم
- لا
- لا اعلم

1. **هل ينتقل مرض كورونا للطفل عبر حليب الام؟**

- صح
- خطأ
- لا اعلم

1. **الطريقة الأفضل للولادة في حال الاصابة بمرض كورونا هي؟**

- ولادة طبيعية
- الولادة القيصرية

1. **هل تنقل العدوى بفيروس الكورونا للجنين وتؤدي لحدوث تشوهات للجنين؟**

- صحيح
- خطأ
- لا اعلم

1. **هل يجب فصل حديث الولادة عن امة المصابة لحين شفائها بشكل تام من المرض؟**

- صحيح
- خطأ
- لا اعلم

1. **العلاجات المستخدمة لعلاج مرض كورونا قد تؤدي الى حدوث تشوهات عند الجنين؟**

- صحيح
- خطأ
- لا اعلم

1. **هل تعتقدي بأنه يجب اسقاط الجنين مباشرة عند الاصابة بمرض كورونا؟**

- صحيح
- خطأ
- لا اعلم

**Perceptions:**

1. **هل توافق على أنه يتم التحكم في كورونا بنجاح ؟**

- أوافق
- لا أوافق
- لا اعرف

1. **هل لديك ثقة في أن الاردن يمكن أن تكسب المعركة ضد فيروس كورونا؟**

- نعم
- لا

1. **يجب علينا الالتزام في المنزل طيلة فترة انتشار الفيروس**

- أوافق
- لا أوافق
- لا أعرف

1. **عند الخروج من المنزل يجب ترك مسافة كافية (2 متر) بين الاشخاص**

- أوافق
- لا أوافق
- لا اعرف

1. **يجب غسل اليدين مباشرة عند العودة الى المنزل:**

- أوافق
- لا أوافق
- لا اعرف

1. **لا يمكن السفر في هذة الفترة**

- أوافق
- لا أوافق
- لا اعرف

1. **يجب على الحكومة عزل المصابين في غرف خاصة في المستشفيات**

- أوافق
- لا أوافق
- لا اعرف

1. **يجب على الحكومة ان تغلق المدارس والجامعت طيلة فترة انتشار المرض**

- أوافق
- لا أوافق
- لا اعرف

Assessment of Practices

خلال أيام الحظر الصحي وتفشي مرض الكورونا، أجب على الأسئلة التالية بنعم أو لا حسب ما ينطبق عليكِ؟

1. **هل ذهبت إلى أمكان مزدحمة؟**

نعم

لا

1. **هل ترتدي كمامة واقية عند مغادرة المنزل؟**

نعم

لا

1. **هل تقومي بغسل يديكِ بشكل جيد بعد ملامسة أسطح أو اشياء غير معقمة؟**

نعم

لا

1. **هل تستخدمي المعقمات والمنظفات بشكل متكرر**

نعم

لا

1. **هل تستنشقين بخار الماء الساخن لتقليل خطر الاصابة**

نعم

لا

1. **هل تقومين بالأستحمام على الاقل مرة في اليوم؟**

نعم

لا

1. **هل تتجنبين لمس العينين، الانف والفم قبل غسل اليدين بالماء والصابون بشكل جيد**

نعم

لا

1. **هل تحرصين على تغطية انفكِ وفمكِ عند العطاس بمنديل او بقطعة قماش**

نعم

لا

1. **هل تحرصين على التخلص من المناديل المستخدمة مباشرةً في سلة الفضلات؟**

نعم

لا

1. **هل تحرصين على التخلص من الكمامة بعد الاستخدام مباشرةً في سلة الفضلات؟**

نعم

لا

1. **هل تعتقدي بأنه إذا ظهرت عليكِ علامات مشابه لعلامات الانفلونزا, يجب أستشارة او مراجعة الطبيب فورا؟**

نعم

لا

1. **هل تحرصين على تناول أغذية ومشروبات صحيه يومياً؟**

نعم

لا

1. **هل تحرصين على ممارسة بعض الأنشطة الرياضة المفيدة داخل المنزل؟**

نعم

لا

1. هل تحرصين على أداء الشعائر الدينية (مثل الصلاة وقراءة القرآن وغيرها) بشكل أكثر من المعتاد؟

نعم

لا

**الجزء الثالث: مقياس الحالة النفسية للمرأة:** يتكون هذا من عشرة فقرات قصيرة تقيس الحالة النفسية للمرأة. أرجو منك الإجابة على جميع الأسئلة التالية وذلك بوضع دائرة حول رمز العبارة التي تصف كيف كان شعورك خلال ***الأيام السبعة الماضية.***

**خلال الأسبوع الماضي:**

**1.كنت قادرة على الضحك ورؤية الجوانب المضحكه في الأشياء**

- لا ينطبق عليّ بتاتاً
- ينطبق عليّ بعض الشيء أو قليلاً من الأوقات
- ينطبق عليّ بدرجة ملحوظة أو بعض الأوقات
- ينطبق عليّ كثيراً جداً، أو معظم الأوقات

**2. أنظر الى الأمور والأشياء حولي بواقعية وبصورة تدعو للتفائل دائماً**

- لا ينطبق عليّ بتاتاً
- ينطبق عليّ بعض الشيء أو قليلاً من الأوقات
- ينطبق عليّ بدرجة ملحوظة أو بعض الأوقات
- ينطبق عليّ كثيراً جداً، أو معظم الأوقات

**3. لُمتُ نفسي بشده و بدون سبب عندما كانت الأمور والأشياء تسير بشكل غير صحيح**

- لا ينطبق عليّ بتاتاً
- ينطبق عليّ بعض الشيء أو قليلاً من الأوقات
- ينطبق عليّ بدرجة ملحوظة أو بعض الأوقات
- ينطبق عليّ كثيراً جداً، أو معظم الأوقات

4**. كُنتُ قلقه بدون أي سبب يدعو للقلق**

- لا ينطبق عليّ بتاتاً
- ينطبق عليّ بعض الشيء أو قليلاً من الأوقات
- ينطبق عليّ بدرجة ملحوظة أو بعض الأوقات
- ينطبق عليّ كثيراً جداً، أو معظم الأوقات

**5. شعرتُ بالذعر والخوف والفزع**

- لا ينطبق عليّ بتاتاً
- ينطبق عليّ بعض الشيء أو قليلاً من الأوقات
- ينطبق عليّ بدرجة ملحوظة أو بعض الأوقات
- ينطبق عليّ كثيراً جداً، أو معظم الأوقات

**6. ازدادت متاعبي وأصبحت الأمور صعبة بشكل يفوق قدراتي**

- لا ينطبق عليّ بتاتاً
- ينطبق عليّ بعض الشيء أو قليلاً من الأوقات
- ينطبق عليّ بدرجة ملحوظة أو بعض الأوقات
- ينطبق عليّ كثيراً جداً، أو معظم الأوقات

**7. كنتُ أجد صعوبة في النوم**

- لا ينطبق عليّ بتاتاً
- ينطبق عليّ بعض الشيء أو قليلاً من الأوقات
- ينطبق عليّ بدرجة ملحوظة أو بعض الأوقات
- ينطبق عليّ كثيراً جداً، أو معظم الأوقات

**8. شعرت بالحزن والتعاسة**

- لا ينطبق عليّ بتاتاً
- ينطبق عليّ بعض الشيء أو قليلاً من الأوقات
- ينطبق عليّ بدرجة ملحوظة أو بعض الأوقات
- ينطبق عليّ كثيراً جداً، أو معظم الأوقات

**9. كنت غير سعيدة لدرجة أنني كنت أبكي كثيراً**

- لا ينطبق عليّ بتاتاً
- ينطبق عليّ بعض الشيء أو قليلاً من الأوقات
- ينطبق عليّ بدرجة ملحوظة أو بعض الأوقات
- ينطبق عليّ كثيراً جداً، أو معظم الأوقات

**10. راودتني أفكار بإيذاء نفسي**

- لا ينطبق عليّ بتاتاً
- ينطبق عليّ بعض الشيء أو قليلاً من الأوقات
- ينطبق عليّ بدرجة ملحوظة أو بعض الأوقات
- ينطبق عليّ كثيراً جداً، أو معظم الأوقات

**اذا اجبت بينطبق على السؤال العاشر يجب مراجعة الطبيب بأسرع ووقت ممكن أو التواصل مع الباحثين على 053903333 فرعي 5452**
