# Supplementary material for: Factors associated with antenatal depression in the Kingdom of Jordan during the COVID-19 pandemic
Source: PLOS Glob Public Health. 2022 Feb 18;2(2):e0000194. doi: 10.1371/journal.pgph.0000194 (PMC10021866; doi:10.1371/journal.pgph.0000194)
Supplement: S3 File — (DOCX) [file pgph.0000194.s003.docx]

**A survey of the knowledge, attitudes and practices of pregnant Jordanian women towards the coronavirus disease (COVID-19) during the outbreak period in Jordan.**

Dear pregnant mother,

In light of the spread of the global epidemic of Corona and the exceptional circumstances we are currently living in, there are many pregnant Jordanian women who suffer from difficulty obtaining information and appropriate medical care during pregnancy. This study will help in assessing these difficulties and study appropriate solutions. Therefore, please fill out this short questionnaire, which takes approximately 10 minutes to complete.

There is no right or wrong answer. The information provided will be used for scientific research purposes only and to derive effective recommendations for improving the health care provided to pregnant women in such exceptional circumstances.

Dear mother, the confidentiality and privacy of the information is preserved, as no information that indicates your personality, such as your name and others, will be taken. Filling out this questionnaire indicates tacit approval to participate in this study and this will not affect you negatively or positively.

With great thanks and appreciation

Dr. Sanaa Abujilban

Faculty of Nursing at the Hashemite University

| **ITEM** | **RESPONSE** |  |
| --- | --- | --- |
| **Are you pregnant?** | **No** |  |
|  | **Yes** |  |
| **If yes, how many weeks?** |  |  |
| **Nationality** | **Jordanian** |  |
|  | Others |  |
| **Demographic information** | | |
| **Age** |  |  |
| **Marital Status** | - Married |  |
|  | - Separated |  |
|  | - Divorced |  |
|  | - Widowed |  |
| **Where do you live?** |  |  |
| **How do you live?** | **I live alone with my husband and children** |  |
|  | I live alone with my children |  |
|  | I Live alone |  |
|  | - I live with my relative in shared house |  |
|  | - Others |  |
| **Education Level** | - Primary School |  |
|  | - Secondary school |  |
|  | - Diploma |  |
|  | - Bachelor |  |
|  | Higher studies |  |
| **How many years did you spend in school?** |  |  |
| **Your Specialty** | Scientific and Health Sciences |  |
|  | Literary and human specialties |  |
|  | Less than high school |  |
| **Your work place** | - I don’t work |  |
|  | - Public |  |
|  | - Private |  |
|  | - Volunteering |  |
| **How many sleeping hours?** |  |  |
| **Do you smoke?** | No |  |
|  | Yes |  |
| **How many Cigarettes per day?** |  |  |
| **Husband age in years** |  |  |
| **What is your husband’s job?** | Not working |  |
|  | Full time Job |  |
|  | Part time job |  |
| **Education of your husband?** | Primary school |  |
|  | Secondary school |  |
|  | Diploma |  |
|  | Bachelor |  |
|  | Higher education |  |
| **Your salary in JD** |  |  |
| **Do you have health insurance?** | No |  |
|  | yes |  |
| **How many pregnancy including the current pregnancy?** |  |  |
| **How many children do you have?** |  |  |
| **Did you have any miscarriage?** | No |  |
|  | Yes |  |
| **If yes, how many?** |  |  |
| **Did you have any health problems before your pregnancy?** | No |  |
|  | Yes |  |
| **If yes, please choose from the following** | - Seizures |  |
|  | - Diabetes Mellitus |  |
|  | - Hypertension |  |
|  | - Heart diseases |  |
|  | - Others |  |
| **Do you have any of the seasonal diseases?** | No |  |
|  | Yes |  |
| **During your pregnancy, did had any health problem?** | No medical problems |  |
|  | Diabetes/ gestational diabetes |  |
|  | - HTN |  |
|  | Heart diseases |  |
|  | twins |  |
|  | Previous abortions |  |
|  | Amniotic fluid problems, amniotic fluid membranes, placental probles |  |
|  | Increase in weight/ obesity |  |
| Do you see your doctor regularly and stick to all appointments during pregnancy or after pregnancy? | No |  |
|  | yes |  |
| How many visits have you made to check on the health of the pregnancy? |  |  |
| Have you avoided or postponed your doctor's visit to you or your child during the quarantine period and the Corona epidemic? | No |  |
|  | yes |  |
| **If yes, how many times did you visit the doctor?** |  |  |
| **Where did you visit the doctor?** | Public hospital/military hospital |  |
|  | University hospital |  |
|  | Private hospital |  |
|  | Private Hospital |  |
|  | Health center |  |
|  | - Others |  |
| Has a health care provider provided you with health information about the Coronavirus, an impact on your health, your child's health, and ways to prevent it during pregnancy or after childbirth? | - No |  |
|  | - Yes |  |
| **If yes, was the information helpful?** | - No |  |
|  | - Yes |  |
|  | - I don’t know |  |
| **Who provide you with the information?** | - **Physicians** |  |
|  | - Midwife |  |
|  | - Nurse |  |
|  | - Others |  |
| **How did they contact you?** | - Care Provider Visit (Dr, Midwife, Nurse) |  |
|  | - Home visit |  |
|  | - Phone call |  |
|  | By the social media |  |
|  | By email |  |
|  | - Others |  |

**Assessment of Knowledge about Corona**

| **ITEM** | | **RESPONSE** |  |
| --- | --- | --- | --- |
| The causes of the disease is Virus | - I don’t Know | |  |
|  | - wrong | |  |
|  | - wright | |  |
| The causes of the disease is Bacteria | - I don’t Know | |  |
|  | - wrong | |  |
|  | - wright | |  |
| **Low immunity increase the possibility to catch the virus** | - I don’t Know | |  |
|  | - wrong | |  |
|  | - wright | |  |
| Corona virus is genetic disease | - I don’t Know | |  |
|  | - wrong | |  |
|  | - wright | |  |
| **Corona virus is infectious disease transmitted from one person to another** | - I don’t Know | |  |
|  | - wrong | |  |
|  | - wright | |  |
| **The Corona virus transmitted through … Please choose one or more correct answer**  The COVID-19 virus spreads via respiratory droplets of infected individuals. | - Air born | |  |
|  | - Blood transfusion | |  |
|  | - Droplets | |  |
|  | Interaction with infected person (touch and kissing) | |  |
|  | Use personal equipment of an infected person? | |  |
|  | Sexual contact | |  |
| The main clinical symptoms of COVID-19 are fever, fatigue, dry cough, shortness of breath, and myalgia | - elevated temperature | |  |
|  | Dry cough | |  |
|  | General weakness | |  |
|  | Difficulty breathing | |  |
|  | Headache | |  |
|  | - Other symptoms (stuffy nose, runny nose, and sneezing, diarrhea, loss of taste or smelling) | |  |
| Unlike the common cold, stuffy nose, runny nose, and sneezing are less common in persons infected with the COVID-19 virus | - I don’t Know | |  |
|  | - wrong | |  |
|  | - correct | |  |
| There currently is no effective cure for COVID-2019, but early symptomatic and supportive treatment can help most patients recover from the infection | - I don’t Know | |  |
|  | - wrong | |  |
|  | - correct | |  |
| . Not all persons with COVID-2019 will develop to severe cases. Only those who are elderly, have chronic illnesses, and are obese are more likely to be severe cases. | - I don’t Know | |  |
|  | wrong | |  |
|  | - correct | |  |
| Eating or contacting wild animals would result in the infection by the COVID-19 virus | - I don’t Know | |  |
|  | - wrong | |  |
|  | - correct | |  |
| Persons with Corona cannot infect the virus to others when a fever is not present | - I don’t Know | |  |
|  | - wrong | |  |
|  | صح correct | |  |
| wearing general medical masks to prevent the infection by the Corona virus | - I don’t Know | |  |
|  | - wrong | |  |
|  | - correct | |  |
| **Wearing plastic or elastic gloves when going out decrease the risk of Corona virus infection** | - I don’t Know | |  |
|  | - wrong | |  |
|  | - correct | |  |
| It is not necessary for children and young adults to take measures to prevent the infection by the COVID-19 virus. | - I don’t Know | |  |
|  | - wrong | |  |
|  | - correct | |  |
| To prevent the infection by COVID-19, individuals should avoid going to crowded places such as train stations and avoid taking public transportations. | - I don’t Know | |  |
|  | - wrong | |  |
|  | - correct | |  |
| **To decrease the risk of Corona infection, I wash hands often after touching things, persons or come back from outside.** | - I don’t Know | |  |
|  | - wrong | |  |
|  | - correct | |  |
| . Isolation and treatment of people who are infected with the COVID-19 virus are effective ways to reduce the spread of the virus | - I don’t Know | |  |
|  | wrong | |  |
|  | - correct | |  |
| People who have contact with someone infected with the COVID-19 virus should be immediately isolated in a proper place. In general, the observation period is 14 days. | - I don’t Know | |  |
|  | wrong | |  |
|  | - correct | |  |
| **How long does it take to appear the symptoms of the disease after the infection?** | Direct after infection | |  |
|  | Maximum after one week | |  |
|  | Maximum in two weeks only | |  |
|  | Maximum in two weeks or more in some cases | |  |
| **Does the Corona virus infection affect the breast feeding?** | - I don’t Know | |  |
|  | - wrong | |  |
|  | - correct | |  |
| **Does the Corona virus transmit through breast feeding?** | - I don’t Know | |  |
|  | - wrong | |  |
|  | - correct | |  |
| **The best birth method is:** | - Cesarean Section | |  |
|  | - Normal Birth | |  |
| **Does the Corona virus transmit to the fetus and cause fetal malformation?** | - I don’t Know | |  |
|  | - wrong | |  |
|  | - correct | |  |
| **Is it necessary to isolate the new born from his infected mother?** | - I don’t Know | |  |
|  | - wrong | |  |
|  | - correct | |  |
| **The medication used to treat Corona can cause fetal malformations** | - I don’t Know | |  |
|  | - wrong | |  |
|  | - correct | |  |
| **Do you believe that you have to abort the fetus direct after Corona infection** | - I don’t Know | |  |
|  | - wrong | |  |
|  | - correct | |  |

**Attitudes:**

| **ITEM** | **RESPONSE** |  |
| --- | --- | --- |
| Do you agree that COVID-19 will finally be successfully controlled? | - I don’t know |  |
|  | - Don’t Agree |  |
|  | - Agree |  |
| Do you have confidence that China can win the battle against the COVID-19 virus? Yes, no Practices | - **No** |  |
|  | - **YES** |  |
| We must stay home during the pandemic infection time | - I don’t know |  |
|  | - Don’t Agree |  |
|  | - Agree |  |
| **We must keep the two meter space when going out** | - I don’t know |  |
|  | - Don’t Agree |  |
|  | - Agree |  |
| **We must wash hands direct when we come back home** | - I don’t know |  |
|  | - Don’t Agree |  |
|  | - Agree |  |
| **We can't travel in this period** | - I don’t know |  |
|  | - Don’t Agree |  |
|  | - Agree |  |
| **The government should isolate infected**  **patients in special hospitals** | - I don’t know |  |
|  | - Don’t Agree |  |
|  | - Agree |  |
| **The government must be ready to close**  **schools if the number of cases increases** | - I don’t know |  |
|  | - Don’t Agree |  |
|  | - Agree |  |

|  |
| --- |

**Assessment of Practices**

| In recent days, have you gone to any crowded place? | No |  |
| --- | --- | --- |
|  | yes |  |
| In recent days, have you worn a mask when leaving home? | No |  |
|  | yes |  |
| Do you wash your hands when you touch dirty things? | No |  |
|  | yes |  |
| **Do you use disinfects and cleaning detergent?** | No |  |
|  | yes |  |
| **Do you inhale vapors to decrease the risk of infection?** | No |  |
|  | yes |  |
| **Do you bath at least once a day?** | No |  |
|  | yes |  |
| I avoid touching the eyes, nose, and mouth | No |  |
|  | yes |  |
| I cover my nose and mouth with a tissue when coughing or sneezing | No |  |
|  | yes |  |
| **I throw the tissue in the trash after I use it** | No |  |
|  | yes |  |
| **I use face mask to cover my nose and mouth in crowded places** | No |  |
|  | yes |  |
| **If you have flu symptoms appeared you should visit the doctor?** | No |  |
|  | yes |  |
| **Do you eat healthy food?** | No |  |
|  | yes |  |
| **Do you do home exercise?** | No |  |
|  | yes |  |
| **Are you keen to perform religious rituals (such as prayer, reading the Qur’an, etc.) more than usual?** | No |  |
|  | yes |  |

**within the last week**

| **Part III:** Please check the answer that comes closest to how you have felt IN THE PAST 7 DAYS, not just how you feel today. | | |  |
| --- | --- | --- | --- |
| ITEM |  |  | |
| **1. I was able to laugh and see funny aspects of things** |  |  | |
| - Does not apply to me at all |  |  | |
| - It applies to me a little or a few times |  |  | |
| - Applies to me to a noticeable degree or some time |  |  | |
| It applies to me very often, or most of the time |  |  | |
| **2. I look at things and things around me realistically and always for optimism** |  |  | |
| Does not apply to me at all |  |  | |
| It applies to me a little or a few times |  |  | |
| Applies to me to a noticeable degree or some time |  |  | |
| It applies to me very often, or most of the time |  |  | |
| **3. I blamed myself for no reason when things and things were going wrong** |  |  | |
| Does not apply to me at all |  |  | |
| It applies to me a little or a few times |  |  | |
| Applies to me to a noticeable degree or some time |  |  | |
| It applies to me very often, or most of the time |  |  | |
| **4.I was worried for no reason** |  |  | |
| Does not apply to me at all |  |  | |
| It applies to me a little or a few times |  |  | |
| Applies to me to a noticeable degree or some time |  |  | |
| It applies to me very often, or most of the time |  |  | |
| **5.I felt panic, fear and dread** |  |  | |
| Does not apply to me at all |  |  | |
| It applies to me a little or a few times |  |  | |
| Applies to me to a noticeable degree or some time |  |  | |
| - It applies to me very often, or most of the time |  |  | |
| **6.My troubles increased and things became more difficult than I could** |  |  | |
| Does not apply to me at all |  |  | |
| It applies to me a little or a few times |  |  | |
| Applies to me to a noticeable degree or some time |  |  | |
| It applies to me very often, or most of the time |  |  | |
| **7.I find it hard to sleep** |  |  | |
| Does not apply to me at all |  |  | |
| It applies to me a little or a few times |  |  | |
| Applies to me to a noticeable degree or some time |  |  | |
| It applies to me very often, or most of the time |  |  | |
| **8.I felt sad and unhappy** |  |  | |
| Does not apply to me at all |  |  | |
| It applies to me a little or a few times |  |  | |
| Applies to me to a noticeable degree or some time |  |  | |
| It applies to me very often, or most of the time |  |  | |
| **9.I was sad and crying too much** |  |  | |
| Does not apply to me at all |  |  | |
| It applies to me a little or a few times |  |  | |
| Applies to me to a noticeable degree or some time |  |  | |
| It applies to me very often, or most of the time |  |  | |
| **10.I had thoughts of hurting myself** |  |  | |
| Does not apply to me at all |  |  | |
| It applies to me a little or a few times |  |  | |
| Applies to me to a noticeable degree or some time |  |  | |
| It applies to me very often, or most of the time |  |  | |

**If your answer applies to the tenth question, you should see a doctor as soon as possible, or contact the researchers**
