# Supplementary material for: Factors associated with antenatal depression in the Kingdom of Jordan during the COVID-19 pandemic
Source: PLOS Glob Public Health. 2022 Feb 18;2(2):e0000194. doi: 10.1371/journal.pgph.0000194 (PMC10021866; doi:10.1371/journal.pgph.0000194)
Supplement: S1 Table — (DOCX) [file pgph.0000194.s005.docx]

| **S1 Table:** Knowledge, Attitudes, Practice and Socio-demographic characteristics with Symptoms of Depression among Jordanian Pregnant Women, showing significant associations, e.g., higher knowledge level was associated with lower depression. | | | | | | |
| --- | --- | --- | --- | --- | --- | --- |
| **Item** |  | **n** | **EPDS mean** | **SD** | **t** | ***P*** |
| COVID-19 knowledge | < 27 | 236 | 12.19 | 6.1 | 2.7 | 0.007** |
|  | ≥ 27 | 310 | 10.8 | 5.7 |  |  |
| COVID-19 attitude | < 8 | 78 | 12.2 | 6.4 | 1.3 | 0.2 |
|  | ≥ 8 | 468 | 11.27 | 5.8 |  |  |
| COVID-19 practice | < 11 | 179 | 11.5 | 5.9 | 0.17 | 0.87 |
|  | ≥ 11 | 367 | 11.4 | 5.9 |  |  |
| Age (*years*) | < 28 | 252 | 11.4 | 6.1 | 0.001 | 0.99 |
|  | ≥ 28 | 294 | 11.4 | 5.7 |  |  |
| Usual sleep duration (*hours*) | < 8 | 221 | 12.6 | 6.3 | 3.96 | .00** |
|  | ≥ 8 | 325 | 10.58 | 5.4 |  |  |
| Household income (*Jordanian dinar)* | < 500 | 229 | 12.04 | 6 | 2.15 | 0.03* |
|  | ≥ 500 | 317 | 10.95 | 5.78 |  |  |
| Education level | High school | 70 | 13.3 | 6.1 | 2.9 | .004** |
|  | Diploma level | 476 | 11.1 | 5.8 |  |  |
| Residence | City | 435 | 11.03 | 5.8 | -3 | .003** |
|  | Village | 111 | 12.89 | 6.1 |  |  |
| Employment | Employed | 204 | 10.7 | 5.6 | -2.3 | .02* |
|  | Housewife | 342 | 11.9 | 6 |  |  |
| Family type | Nuclear | 476 | 11.3 | 5.8 | -1.2 | .2 |
|  | Compound | 70 | 12.2 | 6.3 |  |  |
| Husband’s age (years) | < 32 | 260 | 11.05 | 6.01 | -1.37 | 0.17 |
|  | ≥ 32 | 286 | 11.7 | 5.76 |  |  |
| Husband’s employment | Not employed | 32 | 13.4 | 5.3 | 2 | 0.04* |
|  | Employed | 514 | 11.3 | 5.9 |  |  |
| Husband’s education level | High school | 150 | 12.6 | 5.9 | 3 | 0.003** |
|  | Diploma level | 396 | 10.9 | 5.8 |  |  |
| Number of antenatal visits | < 5 visits | 223 | 10.85 | 5.5 | -1.88 | 0.06 |
|  | ≥ 5 visits | 323 | 11.79 | 6.1 |  |  |
| Gestational age (months) | < 6 | 205 | 10.44 | 5.02 | -3.18 | 0.002** |
|  | ≥ 6 | 341 | 11.99 | 6.28 |  |  |
| Gravida (N) | < 2 | 222 | 11.05 | 5.9 | -1.2 | 0.23 |
|  | ≥ 2 | 324 | 11.66 | 5.9 |  |  |
| Children (N) | < 1 | 231 | 11.02 | 5.8 | -1.3 | 0.19 |
|  | ≥ 1 | 315 | 11.7 | 6 |  |  |
| Miscarriages (N) | < 1 | 405 | 11.23 | 5.8 | -1.14 | 0.26 |
|  | ≥ 1 | 141 | 11.9 | 6.1 |  |  |
| * *p* < 0.05, ** *p* < 0.01 | | | | | | |
