# Supplementary material for: Factors associated with antenatal depression in the Kingdom of Jordan during the COVID-19 pandemic
Source: PLOS Glob Public Health. 2022 Feb 18;2(2):e0000194. doi: 10.1371/journal.pgph.0000194 (PMC10021866; doi:10.1371/journal.pgph.0000194)
Supplement: Attachment — Submitted filename: Rebuttal Table.docx [file pgph.0000194.s006.docx]

Rebuttal Table

| Introduction. 1. This section is too long and unfocussed. For example, consider removing the text about the risk of COVID-19 disease in pregnancy (lines 84-100) and confine the content of the Introduction to psychological aspects of pregnancy in the COVID-19 era and the restrictive measures instituted during national lockdowns. |  |
| --- | --- |
| 2. Line 126…and to examine the effect of KAP toward COVID-19 and socio-demographics on antenatal depression symptoms among Jordanian pregnant women. A cross-sectional, correlational study design was employed. The inherent nature of this research method limits the conclusions that can be inferred about causality; a cross-sectional design can identify associations but can’t examine the ‘effect’ of a variable on the outcome. Please review the stated aim with this in mind and consider rewording to align with the accepted limitation stated in line 327 in the Discussion (Finally, we note the inability to make definite causal inferences due to the cross-sectional design.) |  |
| 3. Lines 135-136, research questions. As above, the study design selected can’t answer the questions posed: Did depressive symptoms in Jordanian pregnant women increase during quarantine in comparison to the reported previous levels? Using an historical comparison is not ideal, primarily because the recruitment and data collection approaches were vastly different (in-person vs online) and online surveys are often not generalisable. For example, pregnant women with moderate-severe depression could be less willing than women with none to engage in an online survey, or could begin but not complete/submit their responses. Conversely, pregnant women with depressive symptoms may be more inclined to be highly interested to engage with online content about mental health or be singled out by their friends during snowballing and encouraged to complete the survey. The population responding to the survey may be very different from the population included in the historical study, so the proportion of women with depressive symptoms observed in this study applies exclusively to the sample of women who completed this survey. Please review all statements made about the hypothesis that depression would ‘increase’ during the lockdown with this in mind (including lines 273-277 in the Discussion). |  |
| 4. Lines 136-137, research questions. Do KAP toward COVID-19 and socio-demographics have an effect on antenatal depression symptoms? As above, the cross-sectional design is able to identify sociodemographic variables associated with depression symptoms but not examine whether they ‘have an effect’. Methods and Materials. |  |
| 5. Consider completing the CHERRIES checklist to ensure all aspects of reporting for online surveys are covered: Eysenbach, G. (2004). Improving the quality of web surveys: the checklist for reporting results of internet e‐surveys (cherries). Journal of medical Internet research, 6(3)e34 doi:10.2196/jmir.6.3.e34 <http://www.jmir.org/2004/3/e34/> The categories requiring additional detail include survey administration, response rates and preventing multiple entries from the same individual. Aspects of the checklist that were not feasible within your study could be mentioned as Limitations. |  |
| 6. Line 187. Analysis. The primary outcome is estimated prevalence of depressive symptoms reported as a percentage. Calculating confidence intervals around that point value would provide more information about its precision. Please consider doing so. |  |
| 7. Line 195. Ethical considerations. This section should cover potential risks and benefits of study participation, as well as ethical oversight, informed consent and confidentiality. Due to the anonymity of the online survey, respondents with depressive symptoms could not be traced to provide care and/or treatment, presumably. Please mention any efforts made by the researchers to enquire if pregnant women engaged with the online survey needed to talk with trained counsellors or to link respondents with available resources to promote wellbeing. |  |
| Discussion. 8. Lines 278-282. The cautionary note in the Discussion about generalisability is well stated. The paper would be strengthened by concentrating on the prevalence observed and the associated socio-demographic and medical factors identified, rather than making comparisons with historical studies of antenatal depression and other populations (frontline health workers) and providing explanations that are pure speculation (line 275-277, 298-300). |  |
| Minor revisions: 9. Line 164. In the current study, the EPDS was found to have a Cronbach’s alpha of 0.85, indicating high reliability. This sentence is confusing because the study being submitted for publication does not involve validation procedures. Please clarify. |  |
| 10. Line 377. This would be likely to increase their adherence to the control measures and lower their levels of antenatal depression. This effect of increasing education and awareness can’t be implied by the data presented; it is possible (vs would be likely) but would need to be assessed. |  |
|  |  |
| Reviewer #2: The article describes an important area area. However, the authors have not described their methodology fully for the reader to understand how they collected their data. It is unclear if the participants had covid infection or they were just pregnant women. |  |
| The use of the term "quarantine" is so unclear here. Do the authors refer to quarantine as those lockdown measures instituted to prevent the spread of covid infection; not necessarily separating a person with covid infection to spread its infection. |  |
| The authors should ensure that they reference their work. They should also revise the way their refence list appears. |  |
